# Role of NF-κB pathway in kidney renal clear cell carcinoma and its potential therapeutic implications

**DOI:** 10.18632/aging.205129

**Published:** 2023-10-16

**Authors:** Jiaao Sun, Feng Chen, Guangzhen Wu

**Affiliations:** 1 Department of Urology, The First Affiliated Hospital of Dalian Medical University, Dalian 116011, China

**Keywords:** KIRC, NF-κB, bioinformatics analysis, prognostic model, treatment

## Abstract

Kidney renal clear cell carcinoma (KIRC), a common malignant tumor of the urinary system, is the most aggressive renal tumor subtype. Since the discovery of nuclear factor kappa B (NF-κB) in 1986, many studies have demonstrated abnormal NF-κB signaling is associated with the development of various cancers, including kidney renal clear cell carcinoma. In this study, the relationship between NF-κB and kidney renal clear cell carcinoma was confirmed using bioinformatics analysis. First, we explored the differential expression of copy number variation (CNV), single nucleotide variant (SNV), and messenger RNA (mRNA) in NF-κB-related genes in different types of cancer, as well as the impact on cancer prognosis and sensitivity to common chemotherapy drugs. Then, we divided the mRNA expression levels of NF-κB-related genes in KIRC patients into three groups through GSVA cluster analysis and explored the correlation between the NF-κB pathway and clinical data of KIRC patients, classical cancer-related genes, common anticancer drug responsiveness, and immune cell infiltration. Finally, 11 tumor-related genes were screened using least absolute shrinkage and selection operator (LASSO) regression to construct a prognostic model. In addition, we used the UALCAN and HPA databases to verify the protein levels of three key NF-κB-related genes (*CHUK, IKGGB,* and *IKBKG*) in KIRC. In conclusion, our study established a prognostic survival model based on NF-κB-related genes, which can be used to predict the prognosis of patients with KIRC.

## INTRODUCTION

Renal cell carcinoma (RCC) is a globally endemic cancer and the sixth most common malignancy in the United States, occurring in approximately 5% men and 3% women in the United States [1, 2]. The most common subtype of RCC is kidney renal clear cell carcinoma (KIRC), accounting for approximately 75% of all kidney cancers. When one kidney is damaged, both kidneys compensate for the damage; therefore, the loss of kidney function is often not detected at an early stage. Therefore, in terms of cancer development, about one-third of RCC patients present with metastases in addition to typical symptoms such as pain, lumps, and hematuria, this limits surgical treatment options [3, 4]. Despite early surgical treatment, up to 30% of patients develop recurrence and metastasis after surgery. Patients with advanced KIRC also have a poor prognosis due to their insensitivity to radiotherapy, chemotherapy, and drug resistance. This results in lower survival rates for patients with KIRC, with 5-year survival rates of only 10–20%. Therefore, owing to the dual difficulties in diagnosis and treatment, there is an urgent need to explore new therapeutic targets and prognostic markers for KIRC [5–7].

NF-κB was first described in 1986 when a “B cytokine” was discovered in the B cells that bind to a site encoding the enhancer region of the immunoglobulin kappa light chain gene and exerts biological effects. Nuclear factor kappa B (NF-κB) is a family of five transcription factors, including NF-κB1, NF-κB2, RelA, RelB, and c-Rel, which share a Rel homologous domain, which is responsible for its binding to DNA [8–10]. NF-κB is mainly present in the cytoplasm of most eukaryotic cells as dimers (p60/p50) and remains inactive in the cytoplasm due to binding to the inhibitory molecule, IκB (inhibitor of NF-κB). In the classical NF-κB-activated pathway, after the cell receives a foreign stimulus signal, IκB kinase in the cytoplasm is activated, IκBα is phosphorylated, and ultimately enabling IκBα to be specifically recognized by E3 ubiquitin ligase for ubiquitination and thereafter specifically recognized and degraded by the 26S proteasome. Then, the p60/p50 dimer is freed and translocated to the nucleus, where it acts as a nuclear transcription factor and participates in regulating the cell transcription process [11, 12]. Activation of NF-κB may be triggered by different signals, such as the binding of a variety of growth factors and cytokines to receptors on the cell membrane, including epidermal growth factor, insulin growth factor, and tumor necrosis factor family members. In addition, the activation of other signaling pathways, such as Ras/MAPK and PI3K/Akt, are also involved in NF-kB activation [13].

The stability of the NF-κB pathway is necessary for cell proliferation, differentiation, and body development. Moreover, the activated NF-κB signal, in addition to participating in cell proliferation and anti/proapoptotic signaling, also maintains the balance of immune regulation in epithelial tissue and inhibits the interference of inflammation with the internal environmental homeostasis of epithelial tissue; increasing evidence also indicates that it plays a key role in the occurrence and development of cancer [14–16]. For example, NF-κB is involved in the proliferation of the breast cancer cell line, T47D, by regulating cyclin D1 and inducing the upregulation of matrix metalloproteases (MMP) 2 and 9; metastasis of liver cancer cell lines, QGY-8024 and PLC97, was increased; and activation of the expression of VEGF, MMP9, and interleukin (IL)-8 in the human prostate cancer cell line, PC-3M, leading to the formation of new blood vessels and invasion of cancer cells. However, such reports in kidney cancer cells are rare [17–21].

In our study, 21 NF-κB-related genes were selected, and the Cancer Genome Atlas (TCGA) database and GSCALite website were used to analyze the copy number variation (CNV), single nucleotide variant (SNV), and messenger RNA (mRNA) expression of these genes in 32 human tumors, as well as their relationship with patient prognosis and anticancer drug sensitivity. Then, Gene Set Variation Analysis (GSVA) cluster analysis was used to divide KIRC patients into three groups to explore the correlation between the NF-κB pathway and occurrence, development, and clinical pathological features of KIRC, aiming to accurately interpret the mechanism of action of the NF-κB pathway in KIRC. Finally, 11 NF-κB-related genes were screened using least absolute shrinkage and selection operator (LASSO) regression to establish a KIRC prognostic model, and the accuracy of the model was further verified at the protein level. These results will guide the clinical diagnosis, treatment, and prognosis of patients with KIRC.

## RESULTS

### NF-κB-related genes are differentially expressed in different cancers and associated with cellular pathways and drug sensitivity

**Figure 1:**
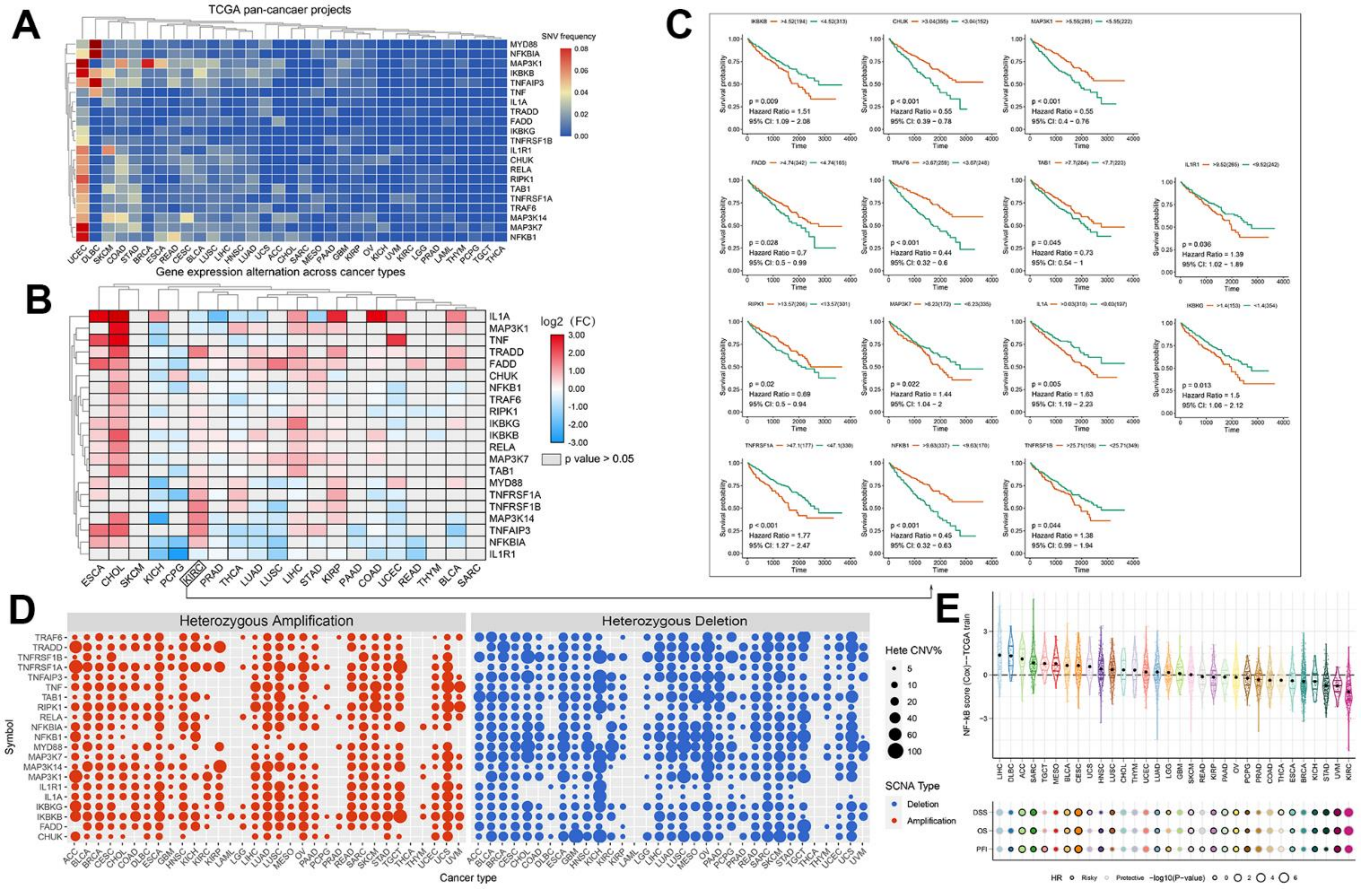
(**A**) SNV frequencies of 21 NF-κB pathway genes in 32 tumor types. Red and blue indicate high and low frequencies, respectively. (**B**) Expression levels of the NF-κB-related genes in 20 cancers. The color code bar shows the corresponding value of log2 (FC) on the right, with values ranging from 3.00 to -3.00 from red to blue. (**C**) Survival curve analysis of all statistically significant KIRC genes in TCGA samples. Red and green represent the high- and low-expression groups, respectively. (**D**) CNV frequencies of the 21 NF-κB pathway genes in 32 tumor types. Red and blue indicate amplification and loss of CNV, respectively. (**E**) Prognostic performance of the 11-gene NF-κB score in 32 types of cancers. The center color of the circle indicates the type of cancer, the color of the circle indicates “Risky/Protective”, and the size of the circle indicates statistical differences.

**Figure 2:**
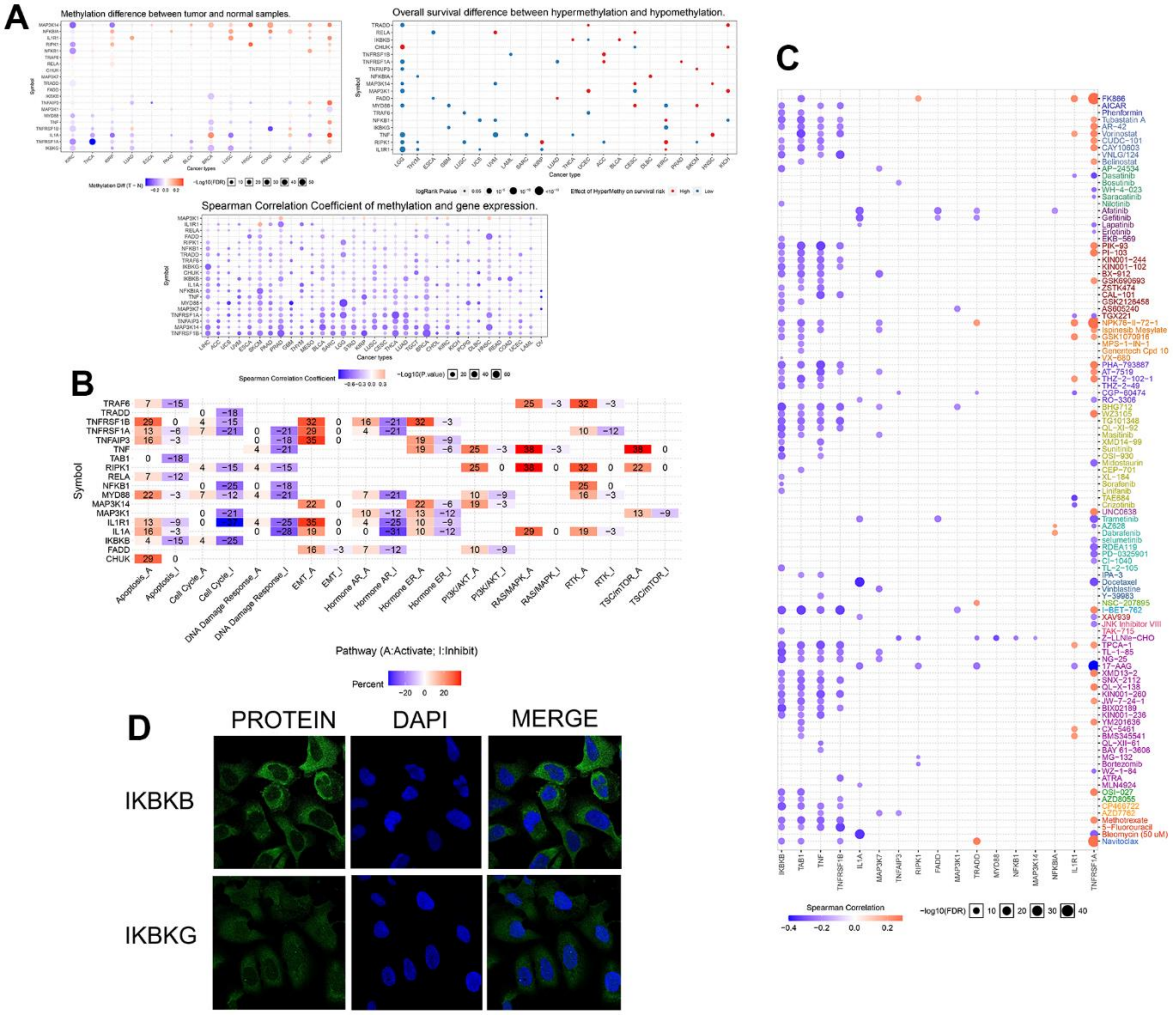
(**A**) The three figure parts show the different degrees of methylation of NF-κB-related genes in several human cancers, the relationship between methylation and mRNA expression level, and the correlation with patient prognosis. The colored circles indicate the value, and the size indicates the relationship with the *p*-value. (**B**) The relationship of NF-κB-related genes to 20 classical cellular pathways, “A” indicates activation and “I” indicates inhibition. (**C**) The relationship between NF-κB-related genes obtained in the GDSC database and drug sensitivity of individual targeted drugs, with blue indicating a positive correlation and orange indicating a negative correlation. (**D**) Immunofluorescence results from the HPA website showed the expression of *IKBKB* and *IKBKG* in the cytoplasm of u2os cells and the staining of the nucleus by DAPI.

To investigate variation and expression changes of NF-κB-related genes in a variety of human tumors, we measured the SNV of NF-κB-related genes in various tumors (Figure 1A and Supplementary Table 1), mRNA expression (Figure 1B and Supplementary Table 2) and frequency of CNV (Figure 1D) based on sample data from the TCGA database. In the mRNA expression profile, we can observe the expression level of NF-κB-related genes in various cancer types; particularly in patients with KIRC, *TRADD*, *MYD88*, *TNFRSF1A*, *TNFRSF1B*, *MAP3K14*, *TNFAIP3*, and *NFKBIA* were higher in KIRC tissues than in normal tissues, and the expression of *IL1A, MAP3K1, CHUK,* and *IL1R1* were lower in the KIRC tissues than in normal tissues. The survival curve of patients with KIRC showed that the expression of most NF-κB-related genes was correlated with patient prognosis (Figure 1C). Such as *CHUK, MAP3KI FADD TRAF6, TAB1* and *RIPK1* raised related to the prognosis of patients with KIRC good genes, and *IKBKB IL1R1, MAP3K7, IL1A, IKBKG* raised KIRC patient’s prognosis related genes. CNV and SNV data obtained from the TCGA database and GSCALite website were analyzed, and the results showed that *TRADD, TNFRSF1A, TNF, MAP3K14, IKBKG,* and *IKBKB* showed CNV amplification in different types of tumors, whereas *TRAF6, TNFRSF1B, TNFAIP3, TAB1, NFKB1, MYD88, MAP3K7,* and *CHUK* showed CNV deletion. From the SNV results, it can be seen that NF-κB-related genes had different degrees of single-nucleotide variation in the 32 tumors. The methylation data of the NF-κB gene set obtained in pancancer through the GSCALite platform showed that the level of NF-κB gene methylation in a variety of cancers differed from that of normal samples, and the expression of *MYD88, TNFRSF1A, TNFRSF1B, MAP3K14,* and other genes had a strong correlation with the level of methylation, and all affected the survival of cancer patients, and the results were statistically different (Figure 2A). The relationship between NF-κB-related genes and classical cellular pathways, such as apoptosis, cell cycle, EMT, etc., showed that NF-κB plays a role in activation or inhibition, such as DNA damage response manifested as a high-intensity activation effect (Figure 2B. The expression of the NF-κB gene is also closely related to drug sensitivity, such as TNF expression is negatively correlated with the sensitivity of various targeted drugs, whereas *TNFRSF1A* is mainly positively correlated (Figure 2C). The u2-os cells are epithelial morphologic cell lines obtained from meso-differentiated sarcomas of the tibia of patients with osteosarcoma; due to the bone metastatic nature of KIRC, we selected the u2-os cell lines for verification, and based on the HPA website, expressions of *IKBKB* and *IKBKG* in u2-os cell lines were found. According to the immunofluorescence results, the target genes, *IKBKB* and *IKBKG*, were clearly expressed in the cytoplasm of the cells (Figure 2D).

### Unsupervised hierarchical clustering and prognostic analysis

**Figure 3:**
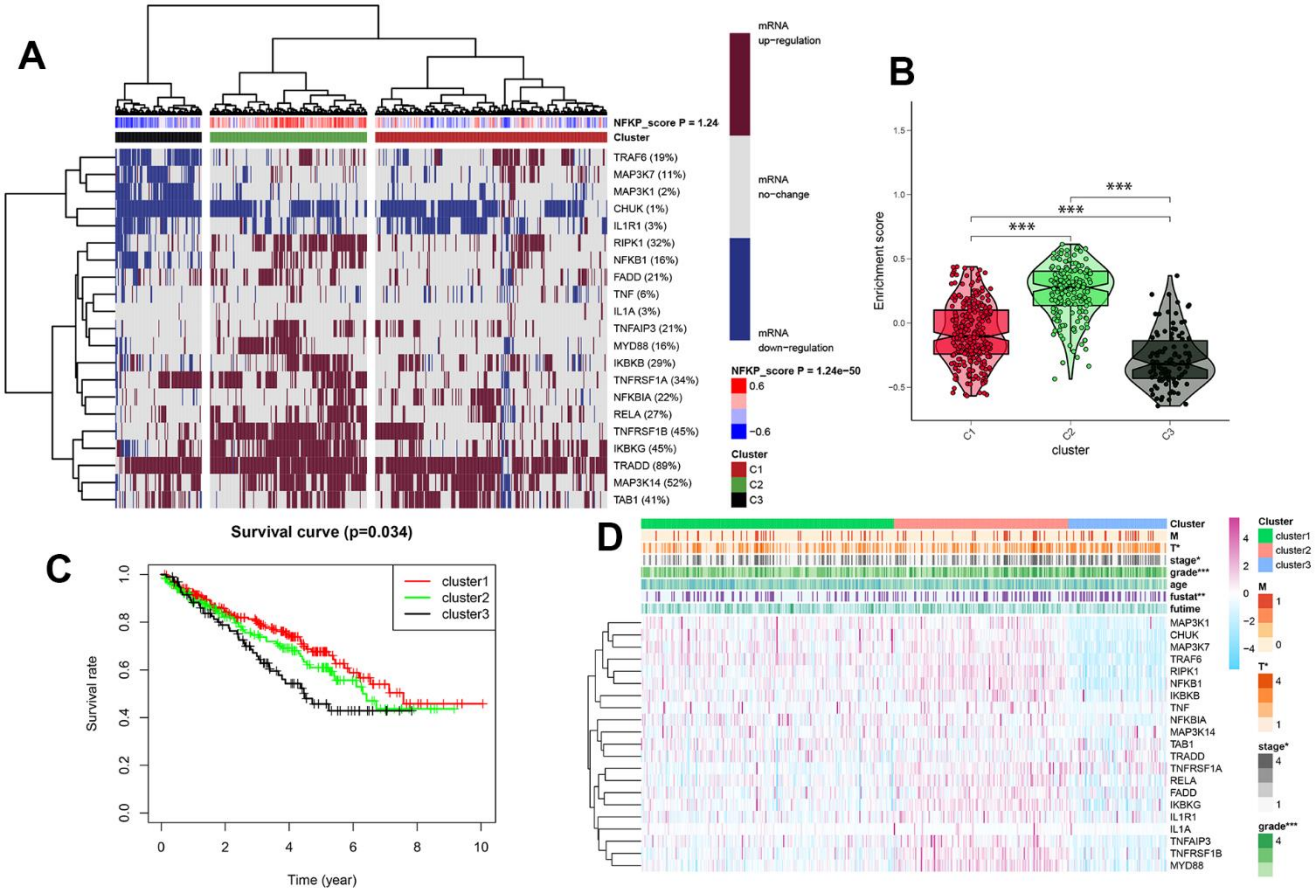
(**A**) All KIRC samples were divided into medium-, high-, and low-expression groups (clusters 1, 2, and 3), according to the level of NF-κB expression, and dark red and blue represent an increase and decrease in mRNA expression, respectively. The redder or bluer the color, the closer the NF-κB score is to 0.4 or -0.4, respectively. Cluster analysis is divided into three groups: red, green, and black for clusters 1, 2, and 3, respectively. (**B**) Enrichment and scores of the three clusters. (**C**) Survival curves for three cluster analyses. (**D**) Clinical pathological features of three clusters of KIRC patients. *: p<0.05, **: p<0.01, ***: p<0.001.

Based on the mRNA expression of the NF-κB-related genes, all TCGA samples of KIRC were divided into three clusters using the GSVA clustering analysis algorithm; the NF-κB score showed different expression levels of NF-κB related genes (Figure 3A), and the resulting C1, C2, and C3 clusters represented normal, active, and inactive scores, respectively. The violin plot (Figure 3B) showed the degree of difference in NF-κB expression between the three clusters, with the *p*-value <0.05, indicating that the difference between cluster groups, obtained by cluster analysis, was statistically significant. The survival curve highlighted the difference in prognosis among patients in the three clusters, indicating that the difference in NF-κB-related gene expression was related to prognosis; combined with the violin diagram results, it showed that patients in the C3 group, whose NF-κB pathway became inactive, had the worst prognosis (Figure 3C). The heat map showed that NF-κB-related gene expression in the three clusters is closely related to patient clinical data and survival outcomes (Figure 3D).

### The NF-κB pathway is closely related to the expression of histone-modified genes and classical oncogenes

**Figure 4:**
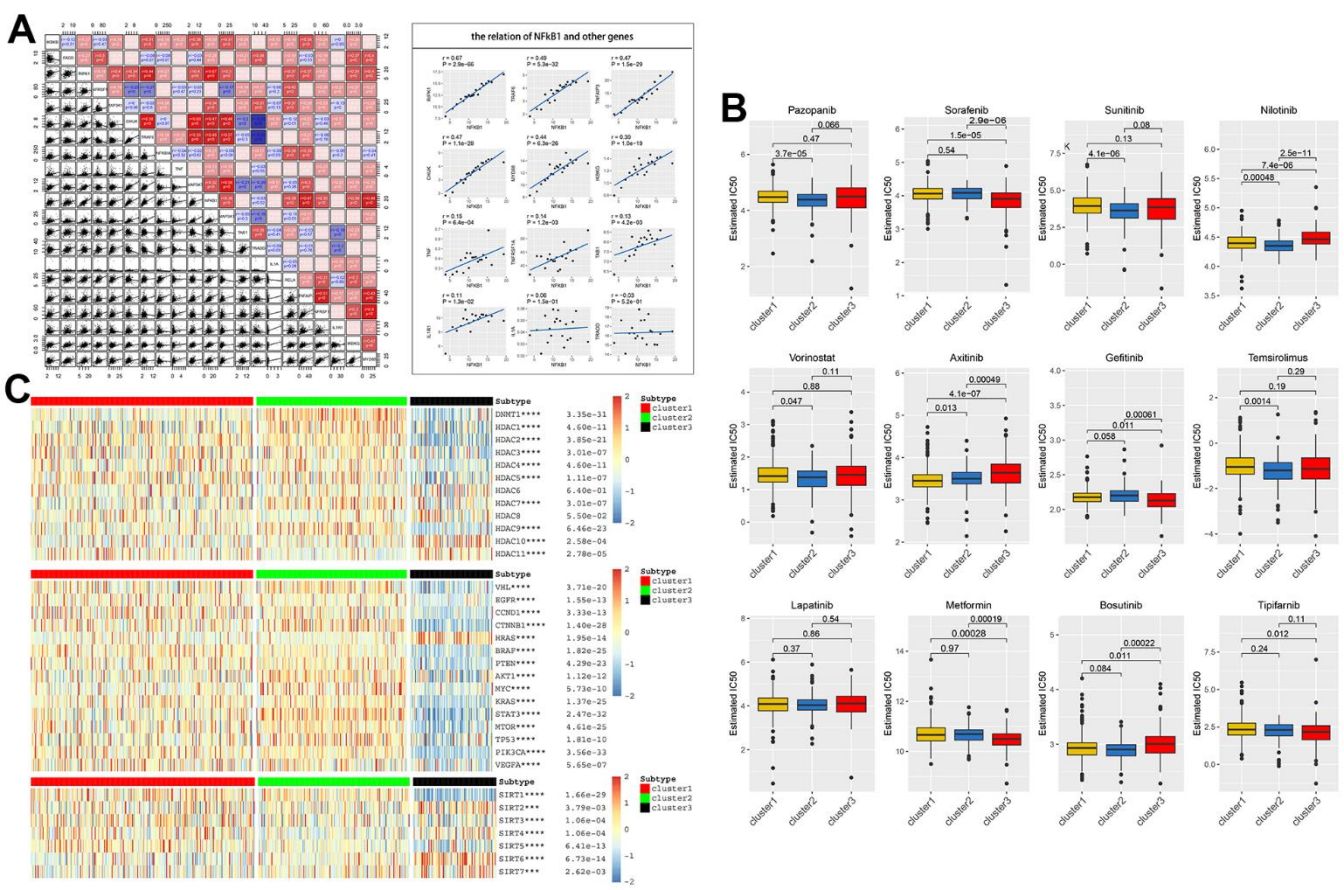
(**A**) Co-expression analysis showed that 21 NF-κB-related genes were associated in tumor tissues, with *R*-value indicating correlation size, red indicating positive correlation, and blue indicating negative correlation, p<0.05 was statistically significant. The regression relationship of NFκB1 with the remaining genes is represented by a scatterplot. (**B**) Based on three clusters, the IC50 predictions of 12 common tumor-targeted drugs for drugs with KIRC cells were analyzed. (**C**) Association of acetylation-related genes (*HDAC* and *SIRT*) and classical tumor family genes with NF-κB scores.

Here, we first explored the relationship between the NF-κB pathway and expression of various classical protooncogenes, for example, the low-expression of *BRAF, PTEN, KRAS, MTOR,* and *PIK3CA* in the NF-κB downregulation group (C3), indicating that the interruption of the NF-κB pathway is related to tumor promotion. The high expression of *CTNNB1, MYC, STAT3, TP53,* etc. in the NF-κB upregulated group (C2) suggested that targeting these genes to treat cancer progression in the NF-κB upregulated group may be effective. In addition, acetylation and deacetylation of transcription factors have also been shown to be associated with a variety of kidney diseases, including diabetic nephropathy [22, 23], the analysis of histone acetylation-related genes showed that abnormal expression of *SIRT* and *HDACs* also had a strong correlation with abnormalities in the NF-κB pathway, the expression of *HDACs* in the NF-κB downregulated group (C3) was slightly lower than that in the other two groups (Figure 4C). Then, to elucidate the correlation between any two genes in the NF-κB pathway, we conducted a co-expression analysis of 21 NF-κB-related genes, and found that the NF-κB1 gene was highly correlated with most other genes in the pathway, such as *RIPK1, TRAF6,* and *TNFAIP3*, and most of the positive correlation. In addition, the correlation between *CHUK* and *MAP3K1*, *TNFAIP3* and *NFRSF1*, *IKBKG* and *MYD88* were also strong (Figure 4A).

### Prediction of the efficacy of KIRC-targeted drugs

To further explore the potential value of the NF-κB pathway in the clinical treatment of patients with KIRC, we plotted box plots based on drug susceptibility predictions in the GDSC database to determine the effect of the NF-κB pathway on IC50 of 12 commonly renal carcinoma targeted drugs. In these drugs, sorafenib is the first targeted multi-kinase inhibitor and first-line chemical drug approved for the treatment of RCC, which blocks formation of tumor neovascularization and directly inhibits the proliferation of RCC cells by blocking RAF/MEK/ERK signaling pathway [24]; Sunitinib inhibits the development of tumor-associated vascular disorders and affects the infiltration of immune cells such as regulatory T cells and macrophages [25]; metformin inhibits RCC cell viability (including cell migration and invasion) and increases apoptosis by disrupting mitochondrial dynamics [26]; other drugs also show different mechanisms of action in cancer suppression. We found that sensitivity to sorafenib, gefitinib, and metformin was higher in the downregulated NF-κB group (C3) but not in the upregulated NF-κB group (C2). In contrast, the sensitivity of the upregulated NF-κB group (C2) to pazopanib, sunitinib, and bosutinib was higher than that of the other two groups (Figure 4B). These results may provide precise guidance for the development of KIRC-targeted drug therapies in the future.

### Immune cell infiltration based on ssGSEA

**Figure 5:**
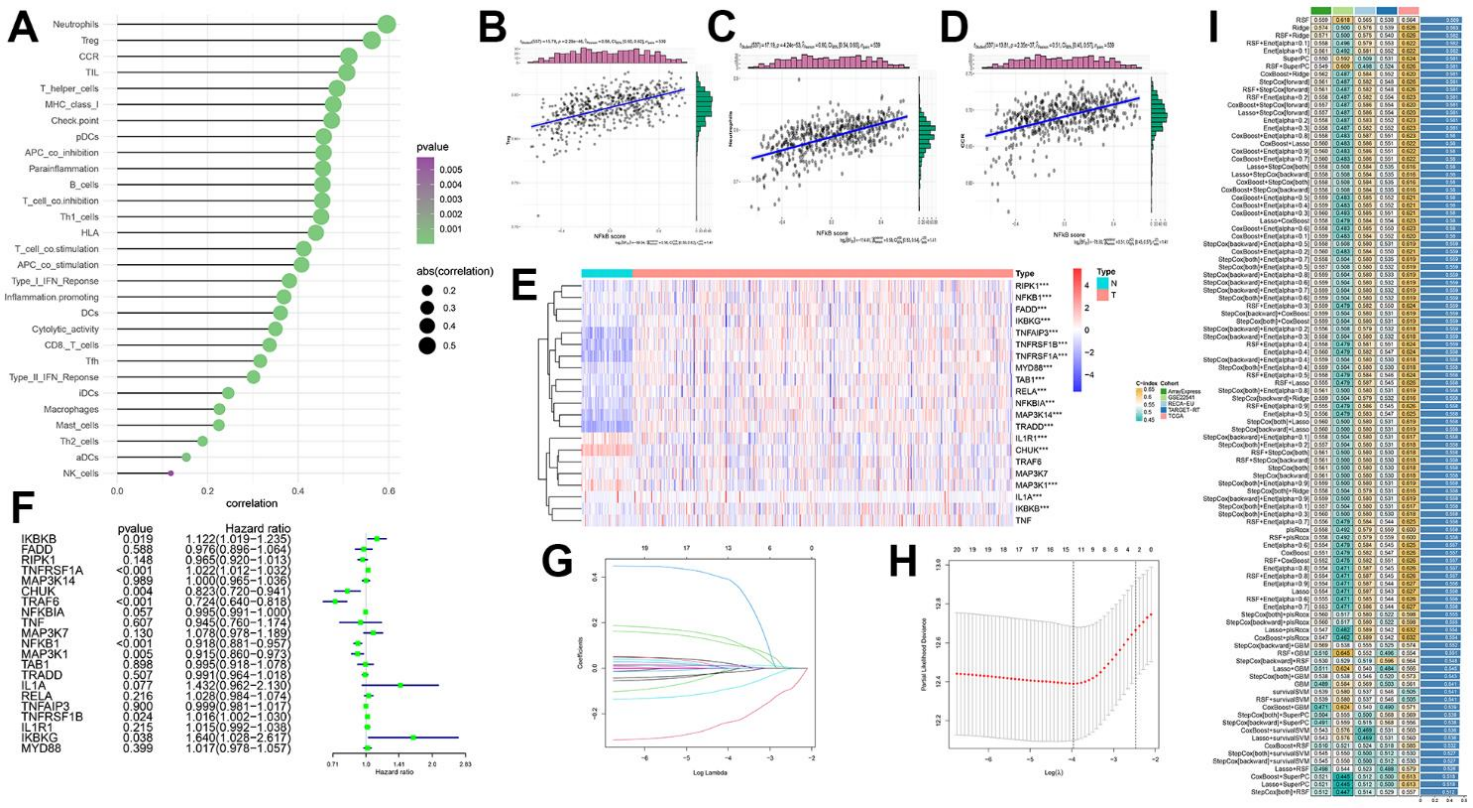
(**A**) Correlation of immune infiltration with NF-κB-related genes. The area of the circle represents ABS (correlation), and the color shows the *p*-value. (**B**–**D**) Scatterplots show the specific relationship between the three immune cells (Treg, neutrophils and CCR) and NF-κB score; they are all positively correlated. (**E**) Expression of NF-κB-related genes between the two samples. Red and blue represent up- and downregulation, respectively, N (green) is the normal sample, and T (red) represents the tumor sample (*: p<0.05, **: p<0.01, ***: p<0.001). (**F**) Forest plot showing hazard ratio analysis and *p*-values for 95% confidence intervals for 21 NF-κB-related genes. (**G**, **H**) LASSO coefficient spectra of NF-κB-related genes in patients with KIRC. LASSO Cox regression analysis was used to screen out the 11 genes. (**I**) Build 101 predictive models and calculate the C-index for each model on all validation datasets.

In addition to targeted drugs, immunotherapy has gradually attracted widespread attention for cancer treatment. To examine the regulatory role of the NF-κB pathway in immunotherapy for KIRC, we investigated the relationship between immune cells and NF-κB. The bubble plot showed a correlation between classical immune infiltration-associated cells or functions and NF-κB (Figure 5A), it can be seen from the results in the figure that most of the immune cells were positively correlated with NF-κB score, and there was a statistical difference. Subsequently, the three immunomodulators most strongly correlated with NF-κB, namely Treg, CCR, and neutrophils, were selected for correlation analysis, and the results were all positively correlated, their correlation coefficients are 5.56, 0.60 and 0.51, respectively, all of them are statistically significant (Figure 5B–5D). Previous studies have also shown that the activity of Treg, CCR and neutrophils is closely related to the occurrence and development of KIRC, which is consistent with the conclusion of our study [27–29].

### Establishing a prediction model using LASSO regression

By analyzing the NF-κB-related gene expression data in the normal control and KIRC groups in the TCGA database, we found that the 21 selected NF-κB pathway genes were significantly different between the two groups (Figure 5E). The figure shows that *RIPK1, NFKB1, FADD IKBKG, TNFAIP3, TNFRSF1B, TNFRSF1A, MYD88, TAB1, RELA, NFKBIA* and *TRRAD* genes expressed in tumor samples is higher. The expression of *IL1R1, CHUK* and *MAP3K1* was higher in normal samples. The forest plot shows the results of the hazard ratio analysis for each gene, which were as follows: *RIPK1, CHUK, TRAF6, NFKBIA, NFKB1, MAP3K1,* and *TRADD* had a protective effect, whereas *IKBKB, TNFPSF1A, MAP3K7, IL1A, RELA, TNFRSF1B, IL1R1,* and *IKBKG* had a risk effect (Figure 5F). We then used LASSO regression analysis to select the appropriate genes to build a predictive model, (Figure 5G, 5H) and selected 11 genes: *TRAF6, NFKBIA, NFKB1, MAP3K1, TRADD, IKBKB, TNFPSF1A, MAP3K7, RELA, TNFRSF1B,* and *IKBKG*. In the TCGA dataset, we fit 101 prediction models through the LOOCV framework, and further calculate the C-index of each model on all validated datasets. If all the samples are paired and their outcomes are compared, the C-index refers to the proportion of combinations where the predicted outcome is consistent with the actual outcome. It can estimate the probability that the predicted results are consistent with the actual observed results, which is used to evaluate the predictive power of the model [30]. The model with the most accurate prediction was random survival forest (RSF), which had the highest average C-index (0.569), and this combined model led the C-index in all validated datasets (Figure 5I).

**Figure 6:**
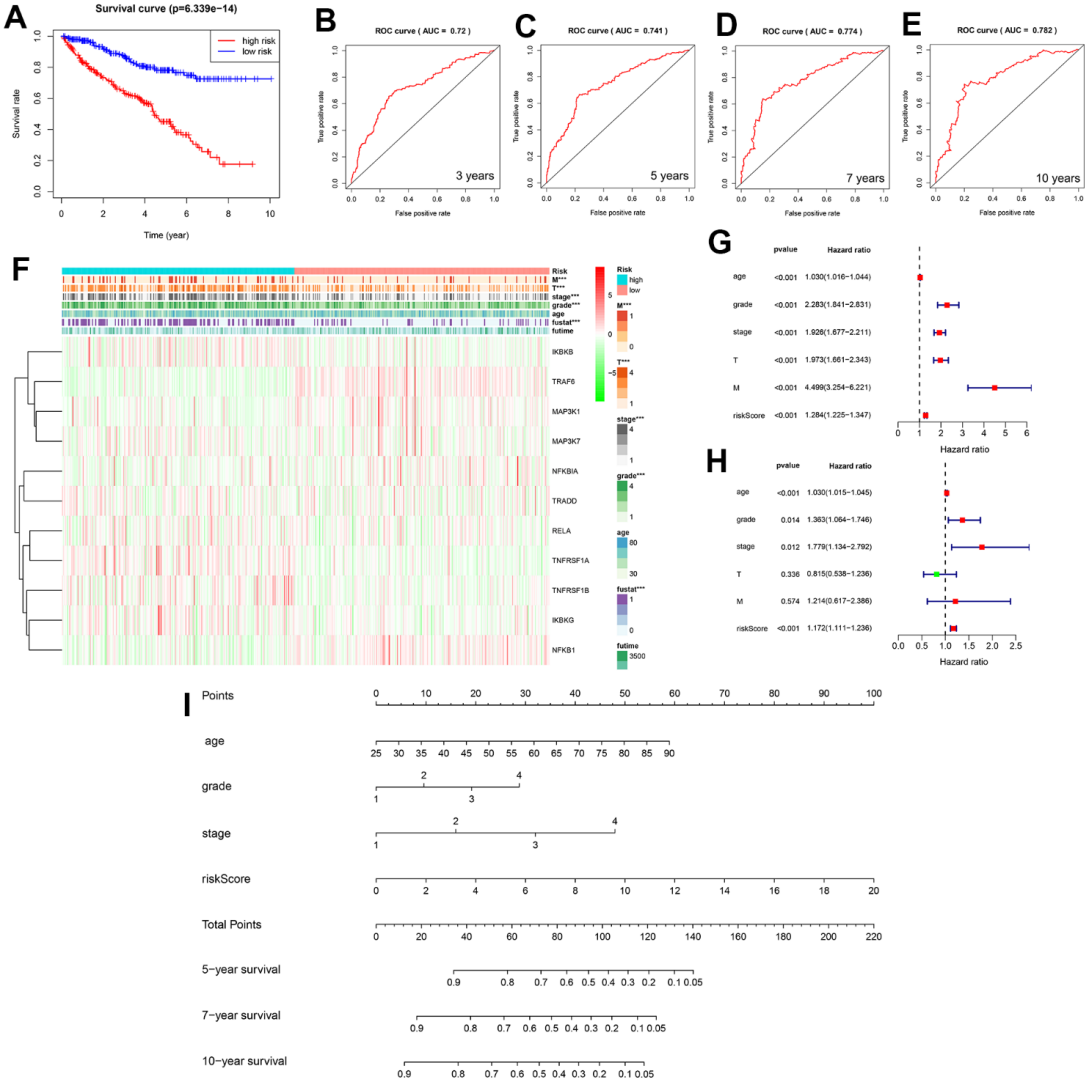
(**A**) Two survival curves based on the model. Blue and red represent the low- and high-risk groups, respectively. (**B**–**E**) Three-, 5-, 7-, and 10-year receiver operating characteristic curves, area under the curve values of 0.72, 0.741, 0.774, and 0.782. (**F**) Heat map showing the correlation between the 11 selected genes and clinicopathological features of the two groups of samples. The two-colored bars indicate gene expression; red and green represent upregulation and downregulation, respectively. (**G**) Forest plot for the univariate Cox regression analysis. (**H**) Forest plot for the multivariate Cox regression analysis. (**I**) Nomogram of the prediction model was used to calculate the total score to obtain the 5-, 7-, and 10-year survival rates of patients with KIRC.

Based on the median risk score calculated from patients with KIRC, patients can be divided into low- and high-risk groups. The survival curves of both groups initially showed the predictive performance of the predictive model (Figure 6A and Supplementary Table 3). ROC curves for four different survival time nodes (3, 5, 7, and 10 years) (Figure 6B–6E) indicated that AUC values for all observed survival time nodes were greater than 0.7 (0.7 and above are considered to have predictive value). To further study the association between NF-κB-related genes and KIRC, we used the Kaplan–Meier “survival” software package to calculate the optimal cut-off value of the risk score, reclassified the cancer patient samples into low- and high-risk groups, and displayed the clinical data and genetic characteristics of the 11 genes from the two groups in the form of heat maps. The results showed that the risk score was strongly correlated with clinical features. The M and T stages, grade, and survival of patients in the high-risk group were worse than those of patients in the low-risk group. *IKBKB, RELA*, and *TNFRSF1A* showed a significant upregulation trend in the high-risk group, whereas *TRAF6* and *NFBIA* showed a significant upregulation trend in the low-risk group (Figure 6F). The univariate Cox regression analysis showed that age, grade, stage, tumor size (T), tumor metastasis (M), and survival model risk scores were associated with Overall Survival (OS) in patients with KIRC; the multivariate Cox regression analysis showed that age, grade, stage, and survival model risk scores were independent risk factors affecting the prognosis of patients with KIRC (Figure 6G, 6H and Supplementary Tables 4, 5).

### Predictive model analysis and validation

We used a nomogram to predict the prognosis of patients with KIRC, and 10 parallel lines were generated; each successive line represented the score, age, grade, stage, and risk score, and the total score calculated by adding the scores of age, grade, stage, and risk score, respectively, after which we could easily estimate the survival rate of patients with KIRC at 5, 7, and 10 years (Figure 6I). The NF-κB scoring system also showed significant organ specificity in the prognosis of different cancers, tumors from the liver, adrenal glands, and testes generally had higher NF-κB scores, while tumors from the uvea, stomach, and breast generally had lower NF-κB scores. Based on the analysis of DSS, OS and PFI, it is suggested that NF-κB-related pathways play different roles in the prognosis of different cancers. The expression of NF-κB in LIHC, MESO, CHOL, and THYM is a protective trend, while the expression of NF-κB pathway in ACC, SARC, SKCM, and UVM is a risk trend (Figure 1E).

**Figure 7:**
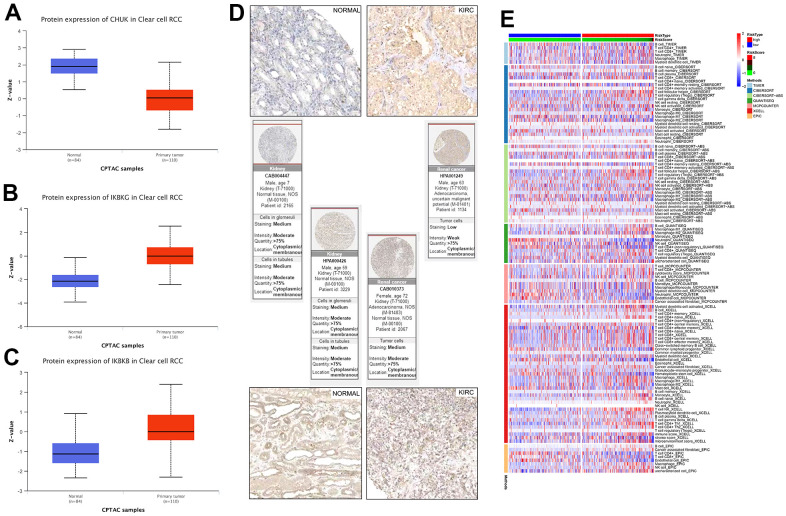
(**A**–**C**) Link between the NF-κB-related genes and KIRC was confirmed at the protein level. (**D**) Immunohistochemical comparison of *IKBKG* and *IKBKB*. (**E**) Based on different algorithms, showing the heat map of the response of immune cells in the high- and low-risk groups. Red and blue represent high and low infiltration levels, respectively, and different algorithms are represented by different colored area bars.

Several additional analyses were conducted to verify the accuracy and validity of the model; we selected three genes for protein level verification, namely *CHUK, IKGGB,* and *IKBKG*. Among them, *IKGGB* and *IKBKG* are protective genes used to build the model, while CHUK is a risk gene associated with the prognosis of KIRC patients but not used to build the model. Firstly, the differential expression of *CHUK, IKGGB,* and *IKBKG* between normal and cancerous tissues was analyzed at the protein level using the UALCAN database, and the results were consistent with the mRNA expression results (Figure 7A–7C). Subsequently, using the HPA database, we downloaded immunohistochemical images of *IKBKG* and *IKBKB* from normal and cancerous tissues and found that their expression of *IKBKG* and *IKBKB* in renal cancer tissues was higher than that in normal kidney tissues (Figure 7D). Finally, we demonstrated the correlation between high- and low-risk scores and the level of immune cell invasion using various immune infiltration algorithms, such as the low-expression of the endothelial cells and CD4+ T cells in the high-risk group in the EPIC algorithm (Figure 7E). These results can be used to identify and characterize the immune microenvironment in patients with different KIRCs and to further evaluate the efficacy of precision therapy, including immunotherapy.

## DISCUSSION

NF-κB family transcription factors play an important role as stressors in the cellular environment, involved in several physiological processes, such as immunity, inflammation, cell proliferation, and death. NF-κB proteins are located in the cytoplasm and can be activated by various stimulus signals. There are two pathways for NF-κB activation, the typical and atypical pathways, and the activated NF-κB translocate to the nucleus, where gene expression is regulated [31]. In terms of cell proliferation and death, NF-κB participates in the expression of cell cycle regulators, including cyclin A, cyclin D1, and CDK6, and the activation of NF-κB can protect cells from TNF-α-induced apoptosis [32]. During cancer development, uncontrolled proliferation and insensitivity to cell death are often accompanied by the activation of NF-κB signaling [33], this is reflected in a variety of cancers, including breast cancer [34], prostate cancer [35], esophageal squamous cell carcinoma [36], lymphoma [37], lung adenocarcinoma [38], etc. Abnormal NF-κB signaling also frequently leads to abnormal changes in tumor resistance, local inflammatory response, and immune microenvironment [38–41].

As the most common subtype of renal cell carcinoma, increased activity of NF-κB in KIRC has been shown to be associated with upregulation of angiogenic markers, and knockout of NF-κB leads to downregulation of IL-6 [42]. Unfortunately, similar studies of NF-kappa B's mechanism in KIRC are currently known to be superficial. In this study, we first analyzed the mutations and expression of NF-κB-related genes in various human cancers by studying the correlation between gene expression and mutations using clinical report information in the TCGA database. To determine whether NF-κB may be a potential target for KIRC therapy, we compared the effects of NF-κB-related genes on prognosis and drug sensitivity in patients with KIRC; interestingly, we found that the genes involved in the NF-κB pathway are both protective and hazardous.

Then, we divided the KIRC samples into three clusters according to the RNA expression levels of NF-κB-related genes: high, normal, and low expression of NF-κB-related genes, respectively. Based on these three clusters, we constructed a survival curve for NF-κB-related genes. We found that patients with downregulated NF-κB pathways had the worst survival rates. In order to confirm the role of commonly used tumor-targeted therapy drugs in the treatment of KIRC, we conducted GDSC analysis, and the results showed that the IC50 of most targeted drugs for KIRC treatment is related to the level of gene expression in the NF-κB pathway, which indicates that the selection of different drugs according to the characteristics of patients can achieve better efficacy or appropriately reduce drug concentrations to reduce the side-effects of drugs. In addition, acetylation and deacetylation are common epigenetic modifications that play a vital role in the formation and development of tumors. By analyzing the expression of classical oncogenes, such as EGFR and mTOR, and acetylation-related genes in the three clusters, we found that the expression of these genes is mainly related to NF-κB pathways, such as S*IRT1, HDAC9, CTNNBI, KRAS,* and *PIK3CA*. Therefore, it may be more efficient to select different targets in different NF-κB characteristic groups.

Tumors infiltrating immune cells (TIICs) are effective targets for drug improvement in tumor therapy [43]. Studies have shown that patients with KIRC have typical characteristics of immunogenic tumors, and TIICs in KIRC, including CD4 T cells, CD8 T cells, natural killer molecules, and dendritic cells, are inhibited to varying degrees, leading to antitumor immune disorders and successful evasion of immune recognition [44]. Additionally, the microenvironment contains a large number of tumor-infiltrating T lymphocytes and cytotoxic T cells that recognize and selectively destroy tumor cells [45]. It has been proved that the number of Treg cell subsets in peripheral blood can help predict the prognosis of immunotherapy, and can reflect the anti-tumor immune status in KIRC patients [46]. In addition, infiltrating neutrophils promote KIRC migration and invasion through VEGFa/HIF2α and estrogen receptor β signaling [47]. Chemokines and their receptor proteins have also been shown to regulate a variety of biological processes [48]. Exploring the relationship between NF-κB and immunity will help us gain a deeper understanding of immunotherapy. Our results show that NF-κB-related genes are closely related to immune cell infiltration, and three of these immunomodulatory factors, Treg, CCR, and neutrophils, are strongly positively correlated with the NF-κB pathway. These analyses will allow patients to receive personalized treatments and provide new ideas for the development of novel targeted therapies.

Finally, 11 genes in the NF-κB pathway were screened out using LASSO regression, namely TRAF6, NFKBIA, NFKB1, MAP3K1, TRADD, IKBKB, TNFPSF1A, MAP3K7, RELA, TNFRSF1B, and IKBKG, among them, IKBKB, TNFPSF1A, MAP3K7, IL1A, RELA, TNFRSF1B, IL1R1, and IKBKG are risk genes for KIRC patients, and RIPK1, CHUK, TRAF6, NFKBIA, NFKB1, MAP3K1, and TRADD are protective genes for KIRC patients. We also constructed a survival prediction model to predict the survival rate of KIRC patients at 5, 7, and 10 years. The area under the ROC curve obtained using the model showed a high predictive value. The multivariate Cox regression analysis revealed that the survival model risk score was an independent risk factor affecting the prognosis of patients with KIRC. The link between NF-κB-related genes and KIRC was confirmed at the protein level. We believe that this model has great value in future clinical research on the treatment and prognosis of KIRC. Specifically, using our survival model and existing clinical data, we can clearly predict the prognosis of KIRC patients. In addition, based on differences in the expression of NF-κB-related genes, each KIRC patient can receive personalized targeted drug therapy, which will greatly reduce the drug resistance of patients and improve the effectiveness of postoperative chemotherapy.

There are few studies on the role of the genes screened in KIRC, all of which are consistent with our conclusions. For example, one study showed that the expression level of *IKBKB* in KIRC is decreased, and the upregulation of *IKBKB* protein levels is associated with an increase in the tumor nuclei grade and a significantly shortened survival period, suggesting that the gene plays a carcinogenic role in KIRC [49]. *RIPK1* is highly expressed in KIRC and is upregulated by TNF-α, which further induces necrotic apoptosis of cancer cells through the RIPK1/RIPK3/MLKL/Drp1 axis [50]. Another study showed that the transcriptional activity of *TRAF6* is regulated by miR146b-5p, and that inhibition of miR146b-5p can increase inflammatory cytokine secretion and *TRAF6* expression in renal tumor mouse models, further inhibiting orthotropic tumor cell growth [51].

However, our study had some limitations. Although we predicted some of the results through bioinformatics analysis, this conclusion lacked support from the experimental data. In other words, although we conducted a preliminary verification of protein levels using the database, we hope that future experiments will further validate the results of our analysis because the role of NF-κB in the pathogenesis of KIRC has not been fully established. In addition, kidney cancer is a cancer prone to metastasis, but the samples we studied lacked lung, liver, brain and other metastatic sites, making this study incomplete. This part will also be an important link in our future research plan.

In summary, our study found that most NF-κB-related genes differ significantly in KIRC expression compared with normal kidney tissue and can be used as risk or protective factors affecting KIRC treatment and prognosis and are closely related to drug sensitivity, immune cell infiltration, classical oncogenes, and histone modification. Classifying patients with KIRC according to NF-κB score is of great significance for evaluating the prognosis of patients and finding new targets, and the prognostic model we constructed may provide more comprehensive suggestions for the development of personalized treatment for patients with KIRC.

## MATERIALS AND METHODS

### Data acquisition and pancancer analysis

The “Biocarta” dataset was found using the Gene Set Enrichment Analysis (GSEA) website (http://www.gsea-msigdb.org/gsea/index.jsp) [52], and 21 genes closely related to the NF-κB pathway were selected for further analysis. The TCGA database is a genome-wide gene expression collection established through large-scale gene sequencing and multidimensional analysis. We used the TCGA database (https://portal.gdc.cancer.gov) to download the changes in SNV and mRNA levels of NF-κB-related genes in 32 cancers [53]. Data were analyzed using Perl, and visual analysis was performed using the “TBtools” software package. Data on CNV, methylation, and classical cellular pathways of genes associated with the 32 cancers were obtained from the GSCALite website (http://bioinfo.life.hust.edu.cn/web/GSCALite/) [54]. Genomics of Drug Sensitivity in Cancer (GDSC) is a publicly accessible database (https://www.cancerrxgene.org/) that provides information regarding drugs, genes, and tumors [55]; based on the GDSC database, we also analyzed the relationship between relevant genes and drug susceptibility of various chemotherapy drugs.

### Cluster analysis based on NF-κB score

GSVA is an analytical method that transforms the expression matrix of genes between different samples into gene sets by unsupervised classification of samples [56]. We used the GSVA algorithm to calculate the NF-κB enrichment score of KIRC patients, and the following three cluster samples were obtained by cluster analysis according to the NF-κB enrichment score of each sample: cluster 1, cluster 2, and cluster 3, which represented the normal, low expression, and high expression of NF-κB pathway-related genes in KIRC patients, respectively. The accuracy of the three clusters was verified with a violin plot and survival curve, and a heat map showing the relationship between gene expression levels and clinical pathological features was generated. A box plot was created using the “pRRophetic” software package in R software to depict the 50% inhibiting concentration (IC50) prediction of the three cluster samples for targeted drugs to treat KIRC [57]. Statistical significance was set at p<0.05.

### Expression of epigenetically-related regulatory genes and classical oncogenes

To explore the different expression patterns of epigenetics and typical oncogenes in the three clusters, we selected two histone acetylation-related genes, deacetylase (SIRT) and histone deacetylase inhibitors (HDACs), as well as classical oncogenes, such as *AKT1* and *mTOR*, analyzed their expression at different levels of NF-κB-pathway activity, and visualized the results in the form of heat maps. Among them, SIRT was involved in important cancer development processes, such as epithelial-mesenchymal transition (EMT), invasion, and metastasis, and plays a significant carcinogenic or cancer-suppressing role [58]. HDACs play an important role in the structural modification and gene expression regulation of chromosomes, and their activities are closely related to the occurrence of cancer and immune diseases [59]. We then used the “corrplot” software package to describe the co-expression relationship between any two NF-κB-pathway genes [60].

### Immune cell infiltration

Single-sample GSEA (ssGSEA) can be applied to gene signals expressed by immune cells in a single sample; we used ssGSEA combined with the expression of relevant genes in the TCGA database to quantify immune cells [61]. Based on the ssGSEA results, we showed correlations between NF-κB scores and 28 types of immune cells, where the area of the spheroids indicated the degree of correlation. Color represents the *p*-value. The R software packages “ggplot2”, “dplyr”, “data.table”, “tidyr”, and “ggstatsplot” were used for analysis and plotting [62–65]. We then selected three classic immunomodulators: regulatory T cell (Treg), chemokine receptor (CCR), and neutrophil and generated scatter plots using the “ggdisterstats” package to show their correlation with NF-κB scores [66].

### Construction of predictive models

We used the “pheatmap” software package to create heat maps to describe differences in NF-κB-related gene expression levels in KIRC and normal tissues [67]. The univariate Cox regression was used to analyze the relationship between NF-κB pathway-related genes and risk indicators (stage, grade, etc.) in KIRC patients. The “glmnet” package was used to construct the LASSO regression model, risk score = ∑Ni = 1 (Expi * Coei); where N, Coei, and Expi were the number of genes, regression correlation coefficient, and gene expression level obtained using LASSO regression analysis, respectively (Supplementary Materials: Table Coef). The cut-off value of the KIRC risk score was calculated using the “survival” package, according to which the samples were divided into high- and low-risk groups, and the survival curve was plotted. Concretely speaking, we calculated the risk score of each sample based on the “survival” package, thus obtaining the median score of all samples, and further divided all samples into high- and low-risk groups according to the median value and the score of each sample. Finally, the receiver operating characteristic (ROC) curve was plotted using the “survival ROC” package to obtain area under the curve (AUC) values for the 3-, 5-, 7-, and 10-year survival rates. AUC is the area value under the ROC curve used to measure the performance of the classifier. The closer the AUC value is to 1, the better the classifier performance, and the closer the AUC value is to 0, the worse the classifier performance. Heat map showed the clinicopathological features of the high- and low-risk groups. Statistical significance level was set at p < 0.05. We also investigated the role of NF-κB-based risk scores in survival of patients with different cancers. We used prognostic models to assess the association of NF-κB scores with disease-specific survival (DSS) in patients with the TCGA pancancer series.

### Validation of the prediction model and nomogram

We also integrated 10 machine learning algorithms and 101 algorithm combinations to develop prognostic models with high accuracy, algorithms include Random survival Forest (RSF), Elastic Network (Enet), Lasso, Ridge, stepwise Cox, and Cox boost, Cox Partial least squares regression (plsRcox), supervised Principal Component (SuperPC), Generalized Enhanced regression modeling (GBM), and survival support vector Machine (Survival-SVM) [68]. Univariate and multivariate Cox regression analyses were used to show the correlation between age, stage, grade, T stage, M stage, and risk scores in the model, with a *p*-value < 0.05 considered statistically significant. RStudio was used for data analysis. To confirm our conclusions, we performed controlled immunohistochemistry experiments on two key molecules involved in the model, *IKBKG* and *IKBKB*, using clinical KIRC specimens. The UALCAN database uses data obtained from TCGA to assess the expression of protein-coding genes and their impact on the survival of patients with 33 types of cancer. The HPA database is a tool based on proteomics, transcriptomics, and systems biology data, which is used to map tissues, cells, organs, etc. [69–71]. We obtained information on protein levels from the HPA (https://www.proteinatlas.org/) and UALCAN databases (http://ualcan.path.uab.edu/). *IKBKB* and *IKBKG* protein immunofluorescence assays were performed using the u2-os cell line. The nomogram was plotted using the “rms” package in R [72].

## Supplementary Material

Supplementary Table 1

Supplementary Table 2

Supplementary Tables 3-5
