# Supplementary material for: Role of NF-κB pathway in kidney renal clear cell carcinoma and its potential therapeutic implications
Source: Aging (Albany NY). 2023 Oct 16;15(20):11313–30. doi: 10.18632/aging.205129 (PMC10637793; doi:10.18632/aging.205129)
Supplement: Supplementary Tables 3-5 [file aging-15-205129-s003.pdf]

Supplementary Table 3. Gene coefficient in prognostic model formula.

Gene	Coef
IKBKB	0.105495885
IKBKG	0.388318763
MAP3K1	-0.009572845
MAP3K7	0.125520029
NFKB1	-0.089029801
NFKBIA	-0.000478901
RELA	0.020199135
TNFRSF1A	0.012994433
TNFRSF1B	0.009781082
TRADD	-0.017761371
TRAF6	-0.236700274

Supplementary Table 4. Univariate Cox regression analysis of the relationship between clinical pathological factors (including risk score) and overall patient survival in the TCGA database.

id	HR	HR.95L	HR.95H	*p*-value
age	1.02970383778191	1.01588047375704	1.04371530010957	0.0000218800056785669
grade	2.28263412688221	1.84053080367777	2.8309325477171	5.72809215140472E-14
stage	1.92576325059956	1.6769942015511	2.21143525358025	1.60470994747149E-20
T	1.97255442351687	1.66095173766084	2.34261530031916	9.64753599422785E-15
M	4.49932539630398	3.25414986954143	6.22095780262227	9.22084574027884E-20
riskScore	1.28439312630962	1.22469433884851	1.34700198292944	6.56558798371527E-25

Supplementary Table 5. Multivariate Cox regression analysis of the relationship between clinical pathological factors (including risk scores) and overall patient survival in the TCGA database.

id	HR	HR.95L	HR.95H	*p*-value
age	1.02973557962054	1.01451867297878	1.04518072676089	0.00011450822245073
grade	1.36288775440025	1.06408584816641	1.74559508924478	0.0142110283383072
stage	1.77943318876541	1.13410284890518	2.79197118350997	0.0121583728705431
T	0.815289621154166	0.537599685077787	1.23641658433175	0.336480414876945
M	1.21364722252324	0.617384208428384	2.38577462887801	0.574463212112357
riskScore	1.17193429101268	1.11116445478337	1.2360276433781	5.22229271568087E-09
